# Transport and retention of laundry microplastic fibres in slow sand filtration systems

**DOI:** 10.1038/s41598-026-41438-x

**Published:** 2026-03-06

**Authors:** Fan Gao, Rosa Busquets, Luiza C. Campos

**Affiliations:** 1 Centre for Urban Sustainability and Resilience, Department of Civil, Environmental and Geomatic Engineering, University College London, Gower St, Bloomsbury, London, WC1E 6BT UK; 2 Faculty of Health, Science, Social Care and Education, School of Pharmacy and Chemistry, Kingston University, Penrhyn Road, Kingston Upon Thames, KT1 2EE UK

**Keywords:** Microfibres, Laundering, Porous media, Sand filter, Water treatment, Engineering, Environmental sciences, Hydrology

## Abstract

This study investigates the transport and retention of microplastic fibres (MFs) in slow sand filtration (SSF) systems using coarse and fine sand media under varying flow conditions. SSF is one of the most energy-efficient and cost-effective tertiary treatment methods. This study examined the performance of SSF for treating washing machine effluents heavily contaminated with MFs, such as those from washing machine discharge. Fine sand filters (with effective grain size of 0.2 mm) consistently retained significantly fewer MFs in the effluent than coarse sand filters (with an effective grain size of 0.6 mm), by a factor of 1.3 ± 0.2, with retention occurring predominantly within the upper 0–10 cm of the filter bed, highlighting the dominant role of the surface layer in MF capture. Between 83.9% and 93.6% of MFs in the filter effluents were 10–50 μm in length, with 10–20 μm fibres forming the dominant fraction across filtration rates of 5, 10, and 20 cm/h. Increasing filtration rate promoted deeper MF penetration into the filter bed, reduced overall retention, and increased MF breakthrough. Dissolved organic carbon removal was slightly higher in fine sand filters than in coarse sand filters, and effluent pH increased modestly with longer retention times. The highest MF removal efficiencies were achieved at the lowest filtration rate (5 cm/h), where removal reached 92.0% and 95.0% for coarse and fine sand, respectively. These trends are consistent with a mechanistic interpretation in which MF transport and retention are governed by interception, straining, and limited diffusion within the porous media, rather than simple size exclusion. SSF therefore represents a promising low-energy treatment option for mitigating MF release from wastewater effluents, particularly for fibres larger than 10 μm.

**Supplementary Information:**

The online version contains supplementary material available at 10.1038/s41598-026-41438-x.

## Introduction

Microplastic fibres (MFs), a dominant type of microplastics (0.1 μm–5 mm), are widely distributed in aquatic, terrestrial, and atmospheric environments^1,2^. Their prevalence is largely attributed to the extensive use of synthetic textiles and fishing materials, which shed fibres through wear and degradation^3^. MFs are increasingly recognised as a risk to ecological and human health^1,2,4^. Notably, over 97.0% of microplastics detected in the human respiratory system are MFs^5^, and polyester MFs (> 3 μm) have been identified in all regions of lung tissue at concentrations of approximately one fibre per gram^6^. Microplastics smaller than 1 μm are categorised as nanoplastics^7–9^, however, their environmental fate remains poorly characterised due to analytical limitations and an incomplete understanding of their transport and retention in engineered treatment systems.

Microplastics can be ingested by biota and undergo translocation across biological membranes, leading to potential physiological effects^10,11^. In addition, microplastics have demonstrated strong adsorption capacities for co-occurring contaminants, including heavy metals^12^, polycyclic aromatic hydrocarbons^13^, and antibiotics^14^. Among these, MFs have shown particular significance in facilitating the transport and distribution of environmental pollutants^15^, owing to their high surface-area-to-volume ratio and persistence in aquatic environments.

MFs enter the environment through multiple pathways, with domestic laundry wastewater identified as a major source^16,17^. A single synthetic garment can release 3,000–465,000 MFs/m² per wash^18^, which are subsequently transported to wastewater treatment plants (WWTPs) via household washing machine effluent. Fibres are frequently reported as the dominant microplastic form across various regions^10,19–22^. To reduce MF loads at the source, France has proposed legislation requiring filters in washing machines^23^. However, source-control measures are not universally implemented, in countries with limited wastewater infrastructure, such as India, Pakistan, and the Democratic Republic of Congo, 63.0%, 92.0%, and 84.0% of municipal sewage, respectively, is discharged untreated into natural waters^24–26^. Even in advanced WWTPs, MFs are not effectively removed. Estimated daily discharges range from 20,000 to 2.1 million MFs, with reports from cities including Plymouth (UK)^27^, Nanjing (China)^28^, Cádiz (Spain)^29^, and Vancouver (Canada)^30^.

Microplastics within the 0.1–500 μm range are considered the most challenging to control^31^. Particles > 500 μm are largely removed during physical and secondary treatment processes^32–34^, whereas smaller microplastics (< 100 μm) remain technically challenging to eliminate due to limitations of conventional treatment technologies^35^. Importantly, reported microplastic removal efficiencies are typically calculated based on particle concentrations rather than mass or volume, making them highly sensitive to changes in particle size distributions and to in situ particle generation during treatment. The elongated and flexible morphology of MFs further complicates their removal, as their long, thin shape allows alignment with flow streamlines and passage through fine filtration media. Consequently, some studies have reported negative MF removal efficiencies, where effluent particle counts exceed influent values (e.g. −15.3%)^36^. Such observations cannot be attributed to poor retention alone but are more plausibly explained by in situ generation of secondary microplastics within treatment processes. Mechanical shear forces associated with rapid mixing, pumping, and aeration can induce fragmentation of larger plastic particles and fibres into smaller microplastics, thereby increasing particle concentrations^37,38^. This mechanism has been experimentally demonstrated in laboratory studies simulating mechanical mixing, which reported substantial plastic fragmentation and the formation of nanoplastics under shear conditions^39,40^. Furthermore, aeration may enhance fibre resuspension and turbulence within treatment units, reducing effective capture efficiency while simultaneously promoting fragmentation, particularly for elongated and flexible fibres such as MFs.

Previous studies into the transport and removal of nanoparticles (solid plastic particles with diameters < 1 μm) in aqueous systems have yielded important mechanistic insights into particle behaviour within porous media^41–43^. Extensive research on engineered nanoparticles and colloids has demonstrated that particle retention in sand and other granular media is not governed solely by pore size exclusion, but rather by a combination of advection, Brownian diffusion, interception, and physicochemical interactions between particles and collector surfaces^44–46^. Nanoparticle removal has been reported to be highly sensitive to solution chemistry, especially ionic strength, pH, and the presence of divalent cations, as these parameters regulate electrostatic interactions and attachment efficiencies^47,48^. Natural organic matter can further enhance nanoparticle mobility by stabilising particles through steric and electrosteric effects, which may promote deeper penetration and breakthrough in porous media^49,50^. More recent studies have extended these findings to nanoplastics, reporting similar transport behaviour and limited retention in sand filtration under unfavourable attachment conditions^41,51^. These studies provide a valuable conceptual framework for interpreting the transport, retention, and breakthrough behaviour of small microplastics and fibres in sand-based filtration systems.

Sand filtration has long been recognised as an effective and accessible technology for mitigating microplastic pollution due to its operational simplicity, low cost, and compatibility with existing water and wastewater treatment systems. Most previous research has focused on rapid sand filtration (RSF), where the removal efficiency of microplastic is strongly influenced by particle size, shape, and filtration conditions. Wang et al.^52^ reported that quartz sand filters removed less than 35.0% of small, micrometre-sized microplastics, whereas larger particles (45–90 μm) were completely eliminated, achieving an overall efficiency of 83.4% for 10 μm polystyrene microbeads. These results indicate that conventional RSF systems are more effective for spherical or granular microplastics, while fibrous forms such as MFs remain more difficult to capture due to their elongated morphology and flexible structure.

Pulido-Reyes et al.^41^ investigated the retention of nanoplastics during conventional drinking water treatment, including sand filtration. Their study simulated slow sand filtration (SSF) using coarse sand media (mean grain diameter 0.450 mm; uniformity coefficient 1.7–1.8) operated at filtration rates of 0.28 and 1.02 m/h. While this work provided valuable insight into nanoplastic transport and retention behaviour, it did not address the removal of MFs, which differ substantially in size, density, and surface properties, leading to distinct pore-scale and hydrodynamic behaviour within porous media.

In wastewater treatment systems, the majority of microplastics are removed during primary and secondary stages. Michielssen et al.^53^ reported that approximately 78.0% of microplastics are captured through sedimentation and flotation in primary treatment, with a further 20.0% removed during secondary biological processes. Tertiary treatment stages such as RSF therefore act primarily as polishing steps. Studies by Bayo et al.^54^ and Tibbetts et al.^55^ demonstrated that RSF can achieve global microplastic removal efficiencies ranging between 64.0% and 75.0%, substantially reducing the abundance of microplastics in treated effluents and receiving waters. Filter design parameters, including grain size, filtration rate, and contact time, are key factors influencing microplastic retention. Wulandari et al.^56^ reported that effectively sized silica sand could remove between 85.0% and 97.0% of larger microplastics, while Wijaya et al.^57^ achieved efficiencies up to 95.0% when combining sand and gravel media.

Despite this progress, the performance of SSF in removing MFs remains poorly understood. SSF offers several unique advantages, including low energy demand, long hydraulic retention times, and the formation of a biologically active surface layer (schmutzdecke) at the top of the sand bed. This layer has been shown to function as a thin, porous, fibrous matrix, characterised by increasing surface area and decreasing porosity resulting from filamentous microbial growth. Previous experimental and modelling studies have demonstrated that schmutzdecke development enhances particle retention by promoting physical straining, interception, and particle attachment within this biologically active layer^58–61^. More recently, the schmutzdecke has been reported to effectively remove fine suspended particles in full-scale drinking water treatment plants, further supporting its role as a key contributor to filtration performance^41^. However, although microplastic removal in sand filtration has been previously investigated, the performance of SSF in removing MFs remains poorly quantified under realistic operating conditions using real laundry wastewater.

Unlike most existing studies that rely on synthetic particles or spherical microplastics, this study focuses specifically on the transport and retention behaviour of microplastic fibres under SSF conditions. Few studies have systematically examined fibre transport, capture, penetration, and release in SSF systems, and current mechanistic interpretations are often extrapolated from non-fibrous particles despite the fundamentally different geometry and transport behaviour of fibres. To address these gaps, the present study systematically evaluates MF transport and retention in SSF systems using fine and coarse sands under varying filtration rates and bed depths. By uniquely integrating effluent-based performance assessment with depth-resolved quantification of retained fibres, this work provides fibre-specific mechanistic insights into capture, penetration, and release processes. This combined approach goes beyond bulk removal metrics and demonstrates the practical potential of SSF as a sustainable, low-energy technology for mitigating MF pollution in wastewater.

## Materials and methods

### Materials

A commercially available 100.0% polyester synthetic fleece blanket (size 1.68 × 2.29 m) was washed, and the wastewater containing MFs was collected from the washing machine outlet. The polyester blanket was chosen because polyester MFs are the most prevalent type of MFs in wastewater, accounting for 96.3% of those found in the influent water in WWTPs^62^. Additionally, its distinct colours (red and blue) facilitated visual discrimination from airborne fibres originating from laboratory clothing and background contamination. A commercial detergent (Ariel) was used to wash the polyester blanket, sourced from a local supermarket in the London area, at a concentration of 0.1 g/L, using tap water (pH 7.7 ± 0.1; turbidity: 0.2 ± 0.1 NTU) supplied by Thames Water for residential use.

The MFs were collected using a domestic front-loading washing machine (Beko, WM5100W, UK) set to the following conditions: small load (45 L), 30 °C wash cycle lasting 30 min; comprising a 10-min wash, a 12-min rinse, and an 8-min spin at 1200 rpm. For each batch, 50 L of washing machine wastewater was collected twice per week into a clean polyethylene container. The container was manually agitated to prepare samples for the subsequent filtration tests. Samples of the washing machine wastewater were collected and analysed daily over the storage period. The results of MF concentrations and average length are shown in Table S1. This monitoring was conducted as a quality control measure to ensure the stability of MF characteristics during storage and to verify that sample handling did not affect the measured concentrations or size distributions. To minimise contamination, white cotton laboratory coats and nitrile gloves were worn throughout the experiments, and their colours were intentionally different from those of the blanket (predominantly blue and red).

### Filtration system

A schematic representation of the experimental setup is shown in Fig. S1 in the Supplementary Materials. Eight polymethyl methacrylate columns were constructed, each with a total height of 70 cm and an inner diameter of 38 mm (cross-sectional area = 11.34 cm^2^). The bottom of each column was packed with 5 cm of pre-washed gravel (2–5 mm) and topped with 50 cm of filter medium consisting of fine sand and coarse quartz sand of 99.8% SiO_2_ purity, with effective sizes of 0.20 mm and 0.60 mm, respectively. The morphology and grain size characteristics of the sand media are presented in Fig. S4, including particle surface features, grain size distributions, and key physical properties relevant to SSF performance.

The effective sizes of the sand are within the usual range recommended for sand filtration^63^. For the sand used in this study, the ratios of the column diameter to the filter media diameter were 190 and 63, respectively, which were sufficiently large to prevent wall effects during media packing^64^. An overflow pipe was installed 5 cm above the filter medium, and a filter effluent pipe was positioned 1 cm from the bottom. The filter effluent pipe included a valve to control the filtration rate. The filters, numbered 1 to 8, contained different media: columns 1–4 were filled with 50 cm of coarse sand (effective size 0.6 mm), and columns 5–8 with 50 cm of fine sand (effective size 0.2 mm), respectively. Columns 1–3 and 5–7 were run with wastewater, while columns 4 and 8 used Milli-Q water, as depicted in Fig. S1.

A peristaltic pump was used to deliver washing machine wastewater into the filters. The quartz sand used as the filter medium was cleaned by sequential soaking in concentrated hydrochloric acid, sodium hydroxide, and 30.0% hydrogen peroxide, each for at least 24 h. Following each treatment, the sand was thoroughly rinsed with ultrapure water (PURITE Select Analyst 320 system, CAT. L300275, UK; filtered through 0.2 μm membrane filters) until a neutral pH was achieved. The sand was then dried overnight at 60 °C and subsequently calcined at 850 °C for 8 h. The cleaned sand was stored under vacuum until further use^65^.

To assess airborne MF contamination in the lab, blank samples were collected using two parallel filtration columns filled with ultrapure water: one containing coarse sand and the other fine sand.

Washing machine wastewater samples from the sand filtration were collected, and turbidity was tested every 24 h, until it was less than 1 NTU, and the removals of both total coliforms and *Escherichia coli* were greater than 99.0%, indicating filter maturation^66^. Wastewater from the washing machine was used in this study, and laundry wastewater contaminated with MFs was filtered through the eight matured filters. The filtration system was operated continuously at room temperature (20.3$\:\pm\:$ 2 °C) at three filtration rates: 5 cm/h, 10 cm/h, and 20 cm/h^67,68^. The filtration rate of all filters was monitored twice a day and adjusted by the regulating valves on the filter effluent tubes when needed. The flowrates were fixed at 0.06 L/h, 0.11 L/h, and 0.23 L/h for 5 cm/h, 10 cm/h, and 20 cm/h, respectively. A minimum water supernatant level was maintained at 1 cm above the sand filter media, and the duration of every filtration run lasted 3 weeks.

Replicate filter effluent samples were collected every 24 h to quantify the target MFs in laundry wastewater samples before and after filtration. Along with the filter effluent samples, pH and conductivity were measured. Concentrations of PO_4_^3−^, SO_4_^2−^, NO_2_^−^, NO_3_^−^, NH_4_^+^, and dissolved organic carbon (DOC) were determined once a week following the procedures described previously^69^.

### Elution experiments

Sand filter samples were collected at different depths of the sand media at the end of the filtration experiment (0–10 cm, 10–20 cm, 20–30 cm, 30–40 cm, and 40–50 cm from top to bottom). This was achieved by dividing the sand filtration columns into five sequential segments along the depth to determine the MFs retained in each section. A photograph of the columns used is shown in Fig.S1.

Each 10 cm sand layer was transferred to a clean glass beaker and rinsed five times with 2.5 L of Milli-Q water pre-filtered through 0.48 μm filter paper (PURITE Select Analyst 320 system, CAT. L300275, UK), with manual stirring using a glass rod to release the MFs retained in the sand filters^70^. The rinsing water was collected and filtered through a 0.45 μm mixed cellulose ester membranes (Millipore, UK) after each washing process^71^.

### Quantification and characterisation of MFs

Samples were pretreated based on a method described in previous studies, with adjustments as needed^72^. First, 100 mL water samples were vacuum filtered through 0.45 μm mixed cellulose ester membranes (47 mm diameter, Millipore, UK), rinsed with 150–200 mL of 30.0% (w/w) hydrogen peroxide (Thermo Scientific Chemicals, Netherlands) into glass beakers, covered with foil and shaken for 4 h in a constant temperature control incubated shaker (IKA, KS 3000 i, Germany) at 60$\:\pm\:$2 $\:{^\circ{\mathrm{C}}}$ for digestion^73^. To further enhance the oxidation reaction of the organic matter in the water samples, 1 mL of FeSO_4_$\:\cdot\:$7H_2_O (Fisher Scientific, UK) was added and left for 6 h at room temperature^74^.

Density separation was then performed using saturated ZnCl_2_ solution (1.6 g/cm^3^) to isolate polyester MFs (density 1.38–1.40 g/cm^3^) by flotation^75,76^. After 6 h of incubation, samples were filtered through 0.45 μm mixed cellulose ester membranes. The membranes were dried in a desiccator and stored in covered glass Petri dishes prior to analysis. MFs were examined using a stereo microscope (Carl Zeiss Discovery V.8, Göttingen, Germany), and images were captured using an Axiocam 305 Colour digital camera. MF concentration and length were quantified using Fiji/ImageJ software. Removal efficiency was calculated based on particle concentrations in influent and effluent samples and expressed as percentages.

### Data analysis

Physical and chemical parameters, including turbidity, pH, DOC, ion concentrations, and MF removal efficiencies under different filtration conditions, were compared using one-way ANOVA. All MF counts and water quality measurements were performed in triplicate. Statistical analyses were conducted using OriginPro 2021, while MF length distributions across depth intervals were analysed using R (v4.3.1) with the tidyverse (v2.0.0), ggvegan (v0.1-0), and ggplot2 (v3.4.2) packages.

## Results and discussion

### Characterisation of polyester microfibres

The polyester MFs released during the laundering process were characterised by widths of 4–10 μm and lengths of 10–130 μm (Table S1), corresponding to length-to-width (aspect) ratios in the range of approximately 2.5 to 32.5^45,77–80^. Blue, red, and transparent cylindrical fibres dominated, with shorter fibres (10–50 μm) occurring most frequently. This length distribution aligns with fragmentation trends reported during domestic washing^81–83^, and the predominance of small, flexible fibres suggests transport and retention behaviour distinct from that of spherical microplastics during filtration.

The effective pore throat sizes of the sand media were substantially larger than the diameters of the investigated MFs but overlapped with their lengths. Fine sand media exhibited a higher frequency of narrow pore throats and slit-like constrictions, whereas coarse sand contained larger, more open pore spaces. For the fine sand (effective grain size 0.20 mm), characteristic pore throat diameters are typically in the range of 6–25 μm^77,84,85^, while coarse sand (effective grain size 0.60 mm) exhibits markedly larger pore throats, typically ranging from 20 to 180 μm^77,79,86^. In contrast, the polyester MFs had diameters of only 4–10 μm but lengths of 10–130 μm, indicating that MF retention is not governed by simple diameter-based size exclusion but instead depends on fibre length relative to pore throat dimensions.

### Wastewater quality during filtration

**Fig. 1:**
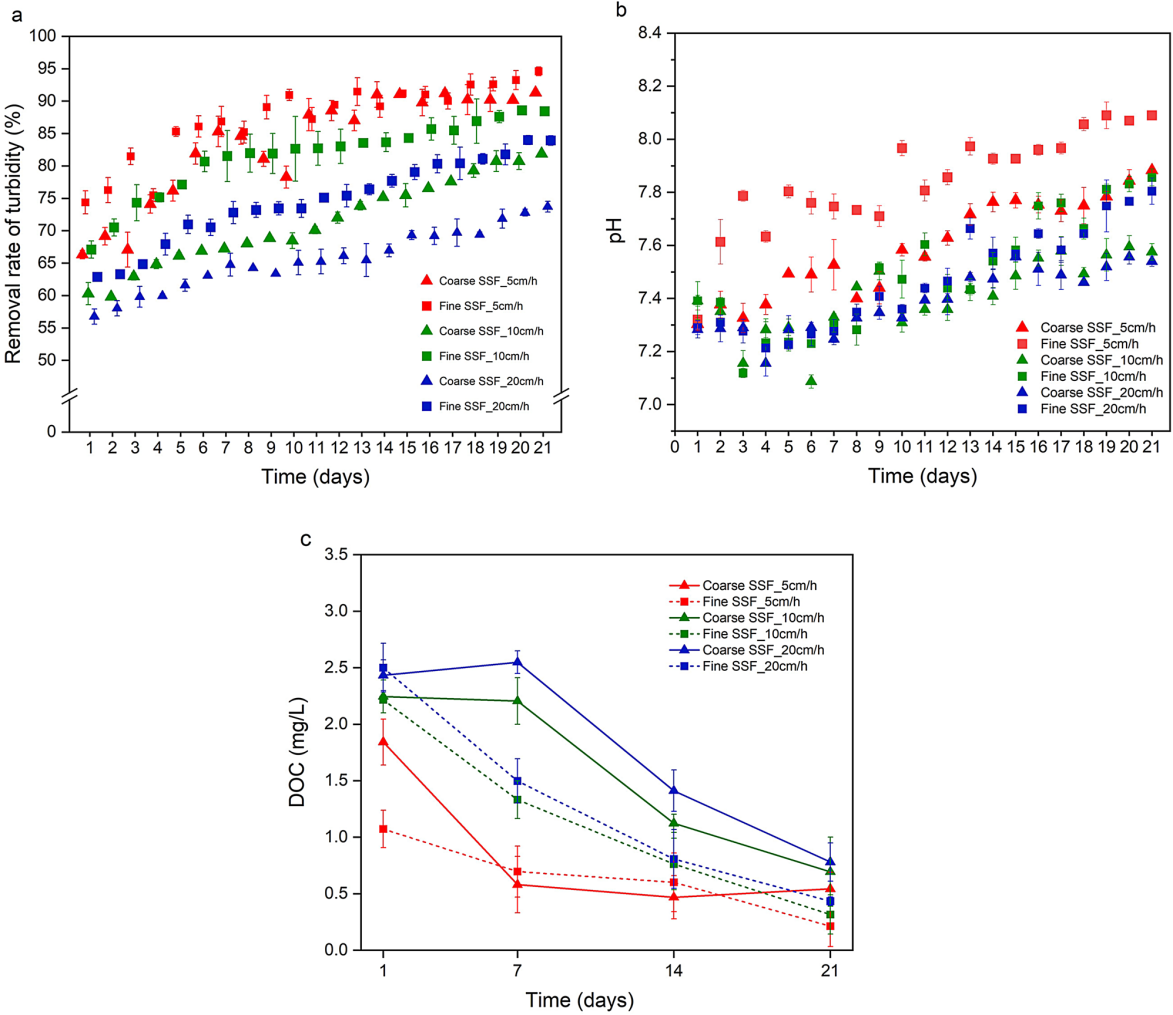
Variation in water parameters (*n* = 3) in slow sand filtration (SSF) system at different filtration rates: (**a**) turbidity, (**b**) pH, (**c**) dissolved organic carbon (DOC) concentrations for coarse and fine sand filtration at three filtration rates. Error bars represent the standard deviation of triplicate measurements.

The turbidity and pH of filter effluents from coarse and fine SSF at filtration rates of 5, 10, and 20 cm/h were monitored, as suspended microplastics and fine particles can influence turbidity and overall water quality^87^. As shown in Fig. 1a, fine sand generally achieved greater turbidity reduction than coarse sand, attributable to its smaller grain size, larger surface area, and improved particle retention, in agreement with Fitriani et al.^88^. Turbidity removal decreased with increasing filtration rate, reflecting reduced contact time and higher pore-water velocities.

Effluent pH (Fig. 1b) was consistently slightly higher in fine-sand SSF than in coarse sand at all filtration rates, likely reflecting enhanced ion exchange and buffering capacity associated with the greater internal surface area of fine media^89,90^. Longer retention time further increased pH because extended water–media contact favours ion exchange and carbonate equilibrium shifts. A comparable rise, from pH 7.0 to 8.5, was reported in sand media reactors by Fitriani et al.^88^. Although these changes were modest, they may be relevant for treatment systems sensitive to minor pH variations.

Retention time and filter media also influenced DOC removal, decreasing concentrations from an average of 3.7 mg/L in the influent to 0.5 mg/L in the effluent. Fine sand, with its smaller pore spaces and larger surface area, enhanced particulate removal and likely supported greater microbial activity and sorptive processes, leading to more consistent DOC reduction, consistent with Attiani et al.^91^. As shown in Fig. 1c, fine-sand SSF showed significantly (*p* ≤ 0.05) higher and more stable DOC removal than coarse SSF across all filtration rates. Longer retention time promotes removal of natural organic matter (NOM) and organic precursors^67^, even in the absence of a visibly developed schmutzdecke. Based on Fig. 1c, average DOC removal efficiencies for coarse and fine SSF were 35.0% and 33.0%, respectively.

The concentrations of major inorganic ions in the raw washing machine wastewater and SSF effluents are summarised in Tables S3 and S4. Fine sand filters consistently achieved greater phosphate removal than coarse sand filters, particularly at lower filtration rates (5 and 10 cm h⁻¹), while nitrate concentrations increased over time in both media, indicating biological nitrification during filter maturation^69^. Sulphate concentrations showed only minor variations across treatments, suggesting largely conservative behaviour. Together, these patterns indicate that fine media and longer retention times favour biological activity and sorptive processes within SSF systems, consistent with progressive filter maturation and schmutzdecke development, which enhance phosphate removal and nitrogen transformations^92^.

### MF concentration and length distribution in filter effluents

In this study, the treated laundry wastewater had pH 7.4 ± 0.1, turbidity 4.5 ± 0.1 NTU, UV-254 absorbance 0.1 ± 0.001, electrical conductivity 593 ± 1 µS/cm, and DO 3.3 ± 0.1 mg/L. MF concentrations ranged from 1,280 to 1,805 MFs/L over multiple washing machine cycles.

**Fig. 2:**
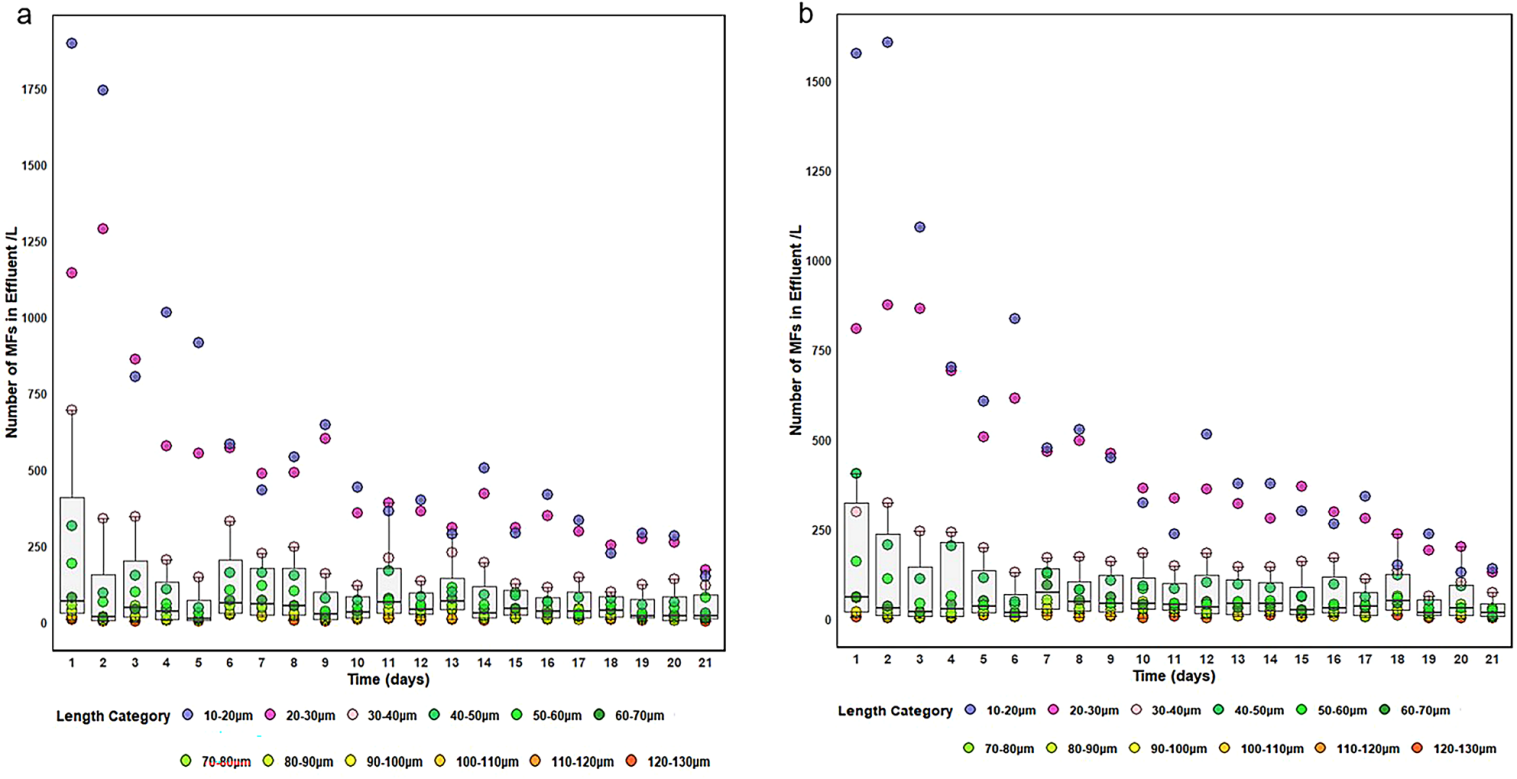
Average concentration of microplastic fibres (MFs) in the filter effluents from (**a**) coarse and (**b**) fine sand filtration at a filtration rate of 5 cm/h. Error bars represent the standard deviation from triplicate measurement (*n* = 3). Dotted data points indicate the size ranges of MFs detected in the effluents.

The concentration of MFs released in the filter effluents from coarse and fine sand SSF, and the corresponding length distributions at 5 cm/h, are shown in Fig. 2. Distributions at 10 and 20 cm/h, representative of rates used in industry, are presented in Figs. S2 and S3. Both filtration rate and sand particle size significantly influenced MF discharge. No MFs shorter than 10 μm were detected in the filtrate, reflecting the optical resolution limit of the stereomicroscope (≈ 7 μm). The influent contained fibres as small as 10 μm, and each filtration run lasted three weeks.

Compared with coarse SSF, fine SSF released 1,564 ± 56 fewer MFs/L during the first five days. MFs 10–30 μm in length showed a marked declining trend over time (Fig. 2). As the filter matured, inter-granular spaces became partially clogged with retained MFs and suspended particles, forming a natural barrier that significantly (*p* ≤ 0.05) enhanced filtration efficiency and improved capture of shorter MFs. Overall, coarse SSF discharged MFs at a rate 1.3 ± 0.2 times higher than fine SSF across all filtration rates. Differences between coarse and fine SSF became more pronounced at higher filtration rates, reflecting the larger porosity and wider pore throats in the coarse sand, which reduced its ability to retain 10–40 μm fibres. In contrast, the narrower pores of the fine sand filter were more effective at trapping MFs, consistent with previous observations for fibrous particles^93^.

MFs measuring 10–50 μm were the dominant size fraction in the effluents from both coarse and fine filters at all filtration rates (Fig. 2). Ziajahromi et al.^94^ reported a similar dominance of short fibres, noting a minimum detectable MF length of 25 μm within this range. Negrete et al.^95^ likewise observed that synthetic fibres longer than 250 μm were retained by sand filters (grain size 1.0 mm) in a conventional DWTP. As filtration rate increased (Figs. S2-S3), MF concentrations in the effluents rose for both media. For example, 1,895 ± 83 MFs (10–20 μm) were detected after one day of coarse sand filtration at 5 cm/h, increasing to 2,157 ± 151 and 2,993 ± 186 MFs at 10 and 20 cm/h, respectively.

The lowest MF concentrations occurred at 5 cm/h for both media, likely due to reduced velocity and extended contact time, which enhance adsorption and interception^96^. In coarse SSF, 10–20 μm MFs accounted for the largest proportion of effluent fibres at all filtration rates. MFs > 50 μm were less prevalent, with fewer than 50 MFs/L exceeding 100 μm detected in coarse-sand effluents at 5 and 10 cm/h, and even fewer in fine-sand effluents. Smaller grain size in the fine SSF created narrower pore spaces that improved sieving and interception; reduced pore throat size lowers the probability of fibre passage, particularly for elongated MFs. Longer fibres are also more prone to tangling and twisting, increasing their likelihood of entrapment^97^.

### Effect of filtration rate on MF removal

**Fig. 3:**
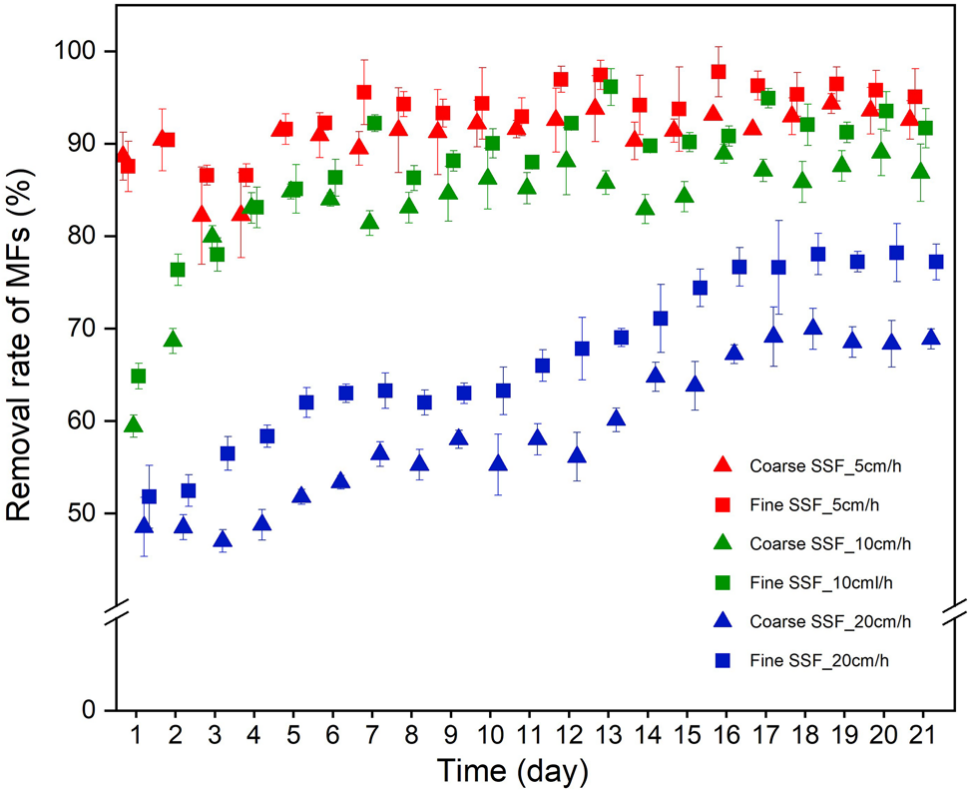
Removal of microplastic fibres (MFs) from washing machine effluent by coarse (effective grain size 0.6 mm) and fine (effective grain size 0.2 mm) sand in slow sand filtration (SSF) systems operated at 5, 10, and 20 cm/h.

Sand filtration proved effective for MF removal from washing machine effluents. At 5 cm/h, removal efficiencies of 92.0% and 95.0% were achieved for coarse and fine filters, respectively, with no significant temporal variation (*p* > 0.05) (Fig. 3). Higher filtration rates led to a decline in performance, particularly in early operation. When the rate increased from 5 to 10 cm/h, removal decreased from 86.0% to 71.0% for coarse media and from 92.0% to 81.0% for fine media.

Filtration rate is a critical design and operational parameter. As expected, slower filtration enhanced MF removal due to longer contact time and extended particle–media interaction^98^. The highest removal rates were observed at 5 cm/h, ranging from 88.6 to 92.6% (coarse SSF) and 87.5–95.1% (fine SSF), with no statistically significant difference between media. At 10 cm/h, all filters maintained removal > 85.0% after 21 days, whereas performance dropped to 70.9% (coarse) and 81.2% (fine) at 20 cm/h (*p* < 0.05). Reduced retention time and increased hydraulic shear at higher rates promote breakthrough of elongated MFs and reduce interception opportunities. ANOVA confirmed that fine sand filter operated at 5 cm/h yielded the most consistent overall performance (*p* < 0.05).

Filtration rate regulates both attachment and detachment of microplastics in filtration systems^41,51^. At lower rates, extended residence time increases the probability of contact and attachment, while lower hydrodynamic shear and re-entrainment forces enhance retention^84^. At higher rates, although collision frequency between particles and collectors may rise, elevated shear and torque acting on retained MFs can induce roll-off and detachment, reducing overall efficiency. This dynamic explains the observed decline at 20 cm/h despite the use of fine sand.

Previous studies on microplastic removal via sand filtration have mostly used synthetic wastewater or broader microplastic classes. In contrast, the present study evaluates MF removal using real laundry wastewater under controlled filtration rates and defined media conditions, thereby providing a more realistic assessment of SSF performance. The results demonstrate that MF removal efficiency depends strongly on both media type and filtration rate, in agreement with existing studies, while offering new insight into fibre-specific transport and retention mechanisms. Wang, Lin & Chen^76^ reported sand filtration (grain size 1.4–2.5 mm) in DWTPs removed 30.9–49.3% of synthetic fibres > 50 μm. Dalmau-Soler et al.^99^ observed 78.0% ± 9 removal for synthetic fibres (20 μm-5 mm) after sand filtration (0.8–1.6 mm) in a DWTP in Catalonia. Dronjak et al.^100^ reported overall removal of 98.3% for synthetic fibres (20 μm-5 mm) after combined treatment including clarifiers, sand filtration, granular activated carbon, and advanced processes.

### Vertical distribution of MFs within the sand bed

**Fig. 4:**
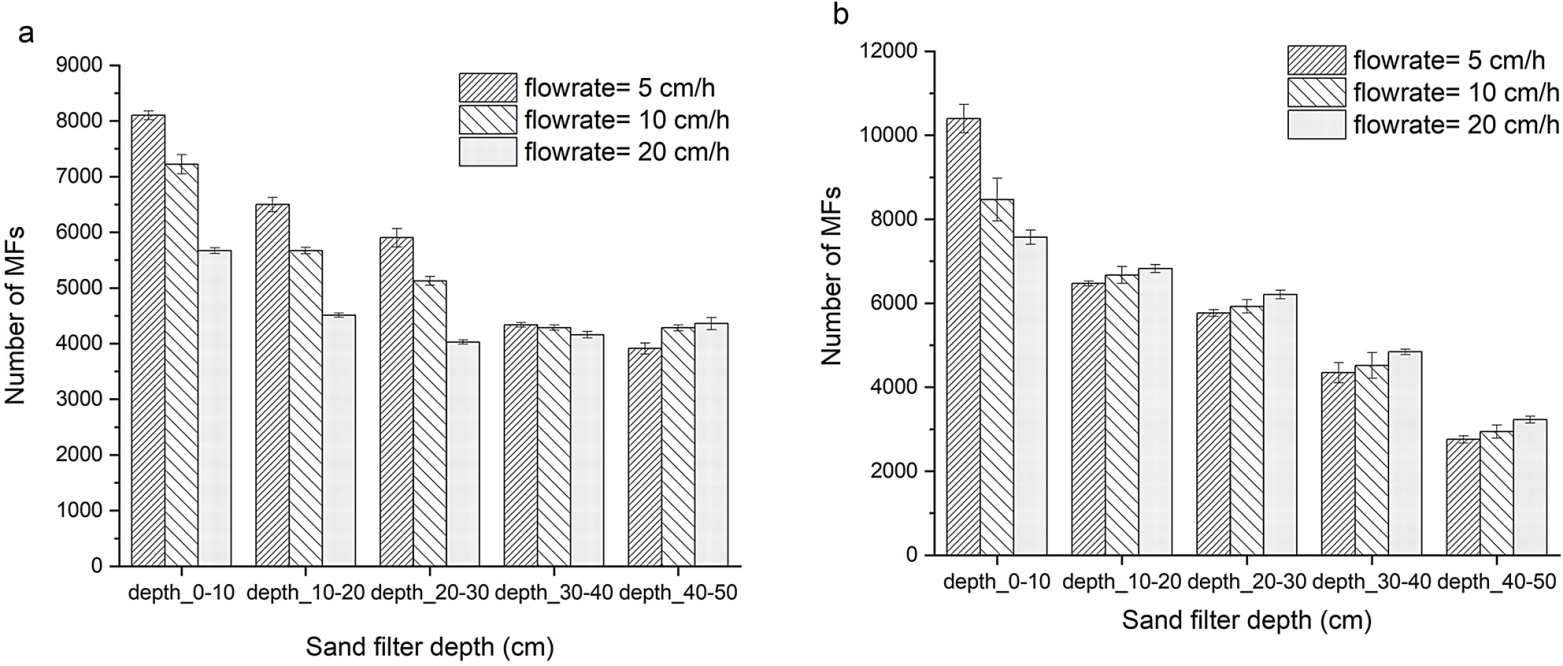
Average penetration of microplastic fibres (MFs) across the depth of (**a**) coarse and (**b**) fine sand filters at filtration rates of 5, 10, and 20 cm/h. Error bars represent the standard deviation from triplicate measurements (*n* = 3).

As shown in Fig. 4, MF penetration depth increased as fibre size decreased, in agreement with Tumwet et al.^101^ and Alhusban^102^. Shorter fibres were therefore able to migrate deeper into the sand bed, whereas longer fibres were preferentially retained near the surface. This has implications for small plastics, including nanoplastics released from treatment works^103^, which are more likely to penetrate deeper into filtration media or bypass retention entirely, posing challenges for effective removal.

These patterns are consistent with studies in which larger microplastics accumulate near the sand surface, while finer particles infiltrate deeper layers^104^. In soil and infiltration systems, microplastics may also act as vectors for co-contaminants, facilitating the downward migration of associated pollutants into subsurface environments^105^. Collectively, these findings indicate that particle size is a primary determinant of penetration depth and spatial distribution, whereas density appears to play a secondary role^105^. Smaller plastic fragments are therefore more readily transported through porous media, while larger fragments preferentially settle and accumulate near the bed surface.

Solution chemistry also influenced MF retention and transport. The observed pH range pH (7.0 ~ 8.5) and DOC (3.66 mg/L) are broadly consistent with conditions under which DLVO-type interactions may affect particle attachment^106^. However, we note that surface interaction energies and zeta potentials were not directly measured, precluding quantitative verification of these interactions. At higher pH or elevated DOC, increased surface charge and steric hindrance can reduce fibre attachment to sand grains, as adsorbed organic matter forms hydrated conditioning films that physically hinder close fibre-collector contact, lowering removal efficiency^50,107,108^. In this study, filtrate pH ranged from 7.0 to 8.5. Quartz sand surfaces are known to become increasingly negatively charged over this pH range due to deprotonation of surface silanol groups (≡ Si–OH → ≡Si–O⁻). Reported zeta potentials typically shift from approximately − 30 mV at pH 7.0 to below − 50 mV at pH 8.0–9.0^109^.

Previous studies have shown that microplastic mobility generally increases as pH rises from 5.0 to 9.0, driven by reductions in hydrodynamic diameter and enhanced Brownian motion^110^. Dong et al.^111^ reported similar trends for PET MFs, with increased mobility increasing at lower electrolyte concentrations, higher pH, and elevated humic acid levels. Although DOC and humic substances are also predominantly negatively charged, their adsorption onto mineral surfaces can generate conditioning films that modify surface roughness and interaction distances, potentially enhancing steric stabilisation while simultaneously increasing opportunities for physical interception rather than electrostatic attachment^112–114^. Intrinsic particle properties, particularly fibre length and aspect ratio, therefore play a dominant role in MF transport behaviour.

In sand soils, the smallest microplastics (~ 21 μm) exhibited the greatest mobility^115^. Engdahl^116^ likewise suggested that shorter fibres bypass low-velocity regions more readily, which may explain why MFs detected at 40–50 cm depth predominantly measured ~ 25 μm in length and ~ 6 μm in width. This corresponds to an aspect ratio of approximately 4, substantially lower than that of longer fibres retained in the upper filter layers. Higher aspect ratios are known to increase interception probability and mechanical entanglement within porous media due to increased rotational constraints and contact area^101,117,118^.

Although aggregation can strongly influence the transport of spherical nanoparticles and microplastics by increasing effective particle size and attachment probability^49,50^, MF transport in porous media is expected to be governed primarily by interception, bending, and mechanical entanglement arising from their elongated geometry and flexibility^15,116^. As a result, fibre-fibre aggregation is likely to play a secondary and transient role under typical filtration conditions, contributing to enhanced retention only when local concentrations or physicochemical conditions favour temporary fibre clustering^119^.

Overall, most MFs accumulated within the upper 0–10 cm of both coarse and fine sand filters at all filtration rates. MF penetration profiles varied with filtration rate. At 10 and 20 cm/h, the distribution in coarse SSF followed: 0–10 cm > 10–20 cm > 20–30 cm > 40–50 cm > 30–40 cm, while the fine sand filter followed: 0–10 cm > 10–20 cm > 20–30 cm > 30–40 cm > 40–50 cm. This variation is attributed to differences in sand porosity, surface roughness, and flow resistance. Decreased porosity and narrower pore throats increase colloid deposition in column systems, and reducing local pore size through finer media enhances MF retention^120^. The measured porosity of unpacked coarse and fine sand columns was 38.6% ± 0.1 and 42.4% ± 0.1, respectively, at a filtration rate of 5 cm/h.

### Mechanistic interpretation

The superior MF removal efficiency of fine-sand SSF across all filtration rates can be attributed to a combination of mechanical filtration mechanisms, surface interaction forces, and time-dependent pore narrowing (ripening). Fine sand has smaller grains, a greater collector surface area, and a higher pore throat frequency per unit depth, which enhances hydrodynamic contact efficiency (η)^110^ and increases the likelihood of fibre-collector encounters.

Upon near-contact, MF retention is governed by a combination of DLVO-type surface interactions and non-DLVO mechanisms, including steric effects, surface roughness, and physical straining. The pH range (7.0–8.5.0.5) and measurable DOC suggest the formation of conditioning films on sand surfaces, which may alter effective interaction distances rather than simply reducing electrostatic repulsion. Once a fibre reaches the collector surface by direct interception or size exclusion, it is more likely to remain attached. Increasing ionic strength reduces electrostatic double-layer repulsion, thereby decreasing particle mobilisation and promoting retention. The observed trends are therefore qualitatively consistent with DLVO theory, while recognising that MF retention in SSF is dominated by coupled mechanical and physico-chemical mechanisms rather than electrostatic interactions alone.

Time-dependent changes in filtration performance further support a ripening-controlled retention mechanism. Accumulation of fibres and suspended particulates in the upper 0–10 cm of the sand bed partially clogs pore spaces, reducing local pore-throat size and increasing surface roughness^51^. This process creates additional straining sites and decreases the probability of fibre detachment. The progressive decline in 10–30 μm fibres in the effluent over time, particularly in fine sand filters, is consistent with ripening-driven enhancement of MF retention.

**Fig. 5:**
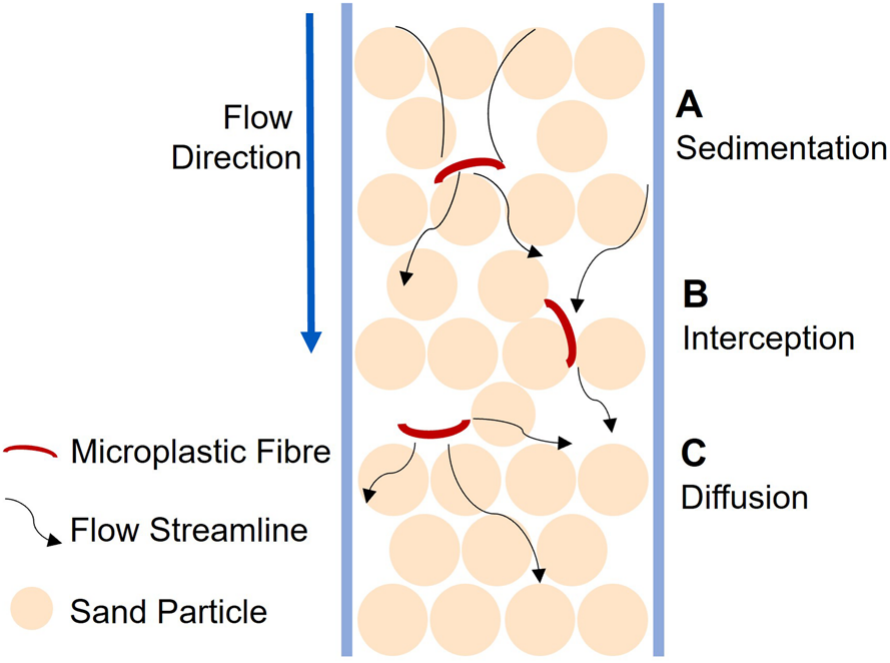
Conceptual schematic illustrating the dominant mechanisms governing microplastic fibre (MF) transport and retention during slow sand filtration. (**A**) Sedimentation, where larger or denser fibres deviate from flow streamlines due to gravitational or inertial forces; (**B**) Interception, where elongated fibres following streamlines contact sand grains as a result of their finite length and proximity to collector surfaces; and (**C**) Diffusion, where smaller fibres undergo Brownian motion and collide with collector surfaces independently of streamline trajectories. The schematic is based on clean-bed filtration theory^44^ and is intended to illustrate the mechanistic interpretation of MF capture inferred from experimental observations rather than direct in situ visualisation.

The dominant mechanisms governing MF transport and retention in the present SSF systems (sedimentation, interception, and diffusion) are summarised conceptually in Fig. 5, which supports the mechanistic interpretation of the experimental results. The combined effects of collector geometry, near-surface attachment, and filter ripening explain the higher MF removal efficiencies observed for fine sand. MF capture mechanisms may be interpreted within the framework of clean-bed filtration theory^44^, which identifies: (A) sedimentation, where larger or denser MFs deviate from flowlines due to gravitational or inertia forces; (B) interception, where elongated fibres following streamlines contact sand grains owing to their finite length and proximity to collector surfaces; and (C) diffusion, where smaller MFs deviate from streamlines via Brownian motion and collide with collectors. Although originally developed for spherical particles, this conceptual framework remains applicable for fibre transport when interception and straining dominate over electrostatic attachment.

MF retention was not governed by simple size exclusion based on fibre diameter, but instead dominated by interception, straining, bending, and mechanical entanglement within the pore network^44,45,116^. Elongated fibres whose lengths exceeded local pore-throat dimensions were more likely to be retained through interception and twisting, whereas shorter fibres were able to align with streamlines and penetrate deeper into the filter bed, particularly at higher filtration rates^46,116^.

According to clean-bed filtration theory, the relationship between influent and effluent particle concentrations is given by:

1 $$\:\mathrm{C}/{\mathrm{C}}_{0}\:=\:\mathrm{e}\mathrm{x}\mathrm{p}\:\left[\frac{-3(1-{\upepsilon\:}){\upeta\:}{\upalpha\:}\mathrm{L}}{2{\mathrm{d}}_{\mathrm{C}}}\right]$$

Where $\:{\mathrm{C}}_{0}$ is the influent particle concentration (mg/L); C is the filter effluent concentration (mg/L); $\:{\upepsilon\:}$ is the bed porosity (dimensionless); $\:{\upeta\:}$ is the transport efficiency (dimensionless); $\:{\upalpha\:}$ is attachment efficiency (dimensionless); L is bed depth (m); $\:{\mathrm{d}}_{\mathrm{C}}$ is the collector (media grain) diameter (m).

Fibre orientation during transport was not directly resolved in this study, and although flow velocity is expected to influence fibre alignment with streamlines, thereby affecting interception and entanglement, the present mechanistic interpretation based on clean-bed filtration theory and effluent-based observations rather than direct in situ measurements of fibre orientation.

**Fig. 6:**
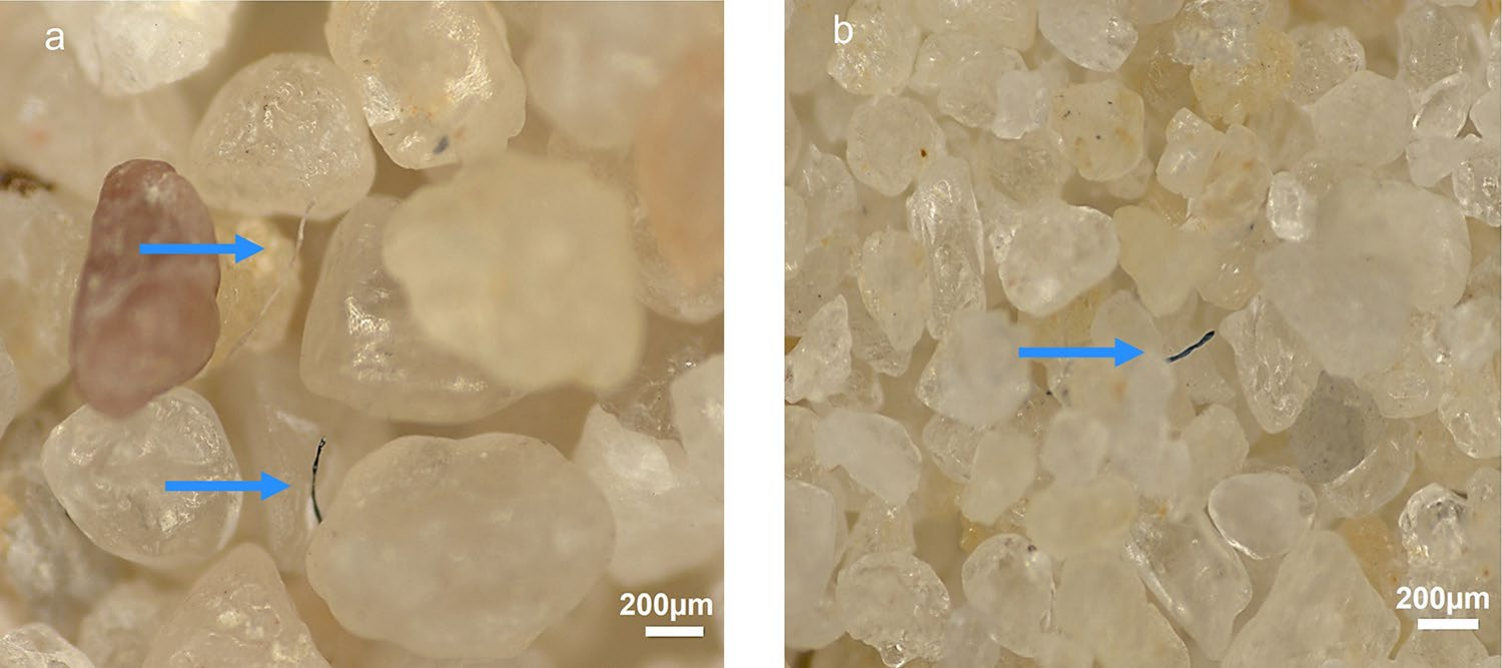
Microfibres (MFs) captured by (**a**) coarse and (**b**) fine sand filters at a depth of 0–10 cm, under a filtration rate of 5 cm/h. Blue arrows indicate the locations of retained fibres.

These mechanistic insights highlight the importance of media selection and hydraulic control in optimising SSF for MF removal. As illustrated in Fig. 6, which compares MF capture pathways in coarse and fine sands, the dominance of interception and straining in fine-sand systems underscores the value of smaller grain sizes and longer residence times, whereas excessive filtration rates promote fibre detachment and breakthrough. In contrast, coarse sand relies more heavily on sedimentation and limited interception, resulting in lower overall capture efficiency and deeper fibre penetration. Maintaining low filtration velocities, well-graded fine media, and progressive ripening is therefore critical for sustained SSF performance.

### Practical implications

This study highlights the operational advantages and limitations of fine sand in filtration systems, particularly for MF retention. Fine sand offers a clear advantage over coarse sand for MF removal, as its smaller grain size creates reduced pore spaces that enhance mechanical straining and promote more consistent surface-level retention^119^. Its greater surface area also supports microbial colonisation and adsorption processes, which may assist in the degradation of co-occurring organic pollutants. Although biological processes such as biofilm development were not directly examined in this study, and no visible schmutzdecke formed during the experimental period, these processes are expected to play an important role in enhancing filtration efficiency^67^ and will be investigated in future work.

All experiments were conducted under downward-flow conditions, consistent with conventional SSF operation, where particle accumulation occurs primarily within the upper sand layer and filter cleaning is achieved by surface scraping rather than backwashing. Consequently, the potential influence of alternative flow orientations (e.g. up-flow filtration) was not examined. Previous studies have reported higher particle deposition rates under up-flow compared with down-flow configurations in porous media^121^, suggesting that flow orientation may be an important design consideration for other filtration systems and warrants further investigation beyond the scope of SSF-focused applications.

Despite its advantages, fine sand also presents operational challenges. Its lower permeability increases hydraulic resistance, potentially limiting filtration rates and necessitating more frequent maintenance to prevent clogging^122^, particularly in systems treating greywater or raw wastewater with high particulate loads^123^. In large-scale or high-throughput applications, this may require additional operational controls or energy inputs to maintain stable flow conditions.

MFs predominantly accumulated within the upper 0–10 cm of the sand bed (26.8% ± 1.7 and 30.3% ± 3.4 for coarse and fine SSF, respectively), with direct implications for filter maintenance. In conventional practice, this fouled surface layer is periodically scraped and washed prior to reuse or replacement. This operation presents a potential secondary pollution pathway, as the wash water may contain concentrated MFs. Surface accumulation of MFs may also promote clogging and progressively reduce filtration efficiency, potentially allowing smaller or detached fibres to pass into the effluent. We observed that the average concentration of MFs released during rinsing of the upper 0–10 cm sand layer at the end of the filtration period, for both filters, was slightly lower (by approximately 4.9%) than the concentration measured in the raw washing machine wastewater (Table S2). These findings highlight the importance of appropriate management and treatment of sand-cleaning wash water generated during filter maintenance, since fibres released during the washing of scraped sand could otherwise represent a secondary source of microplastic pollution.

To mitigate these risks, treatment of sand wash water, for example, via membrane filtration or flocculation, should be considered prior to discharge or reuse. Operators should also establish clear waste-handling protocols to prevent the reintroduction of captured MFs into the environment.

While fine sand provides superior MF retention and enhances overall filtration performance, its application must be balanced against hydraulic efficiency, maintenance frequency, and responsible waste management. Future SSF designs should integrate media optimisation with wash-water recovery and treatment strategies to ensure environmentally sustainable and regulatory-compliant microplastic control.

## Conclusion

This study demonstrates that SSF is an effective and sustainable process for the removal of MFs while simultaneously improving the physico-chemical quality of washing machine effluents across a range of filtration rates. At the lowest tested filtration rate (5 cm/h), MF removal efficiencies reached 92.0% (coarse sand) and 95.0% (fine sand), decreasing to 71.0% and 81.0%, respectively, at the highest rate (20 cm/h) due to reduced residence time and increased hydraulic shear. Across all experiments, the fine SSF consistently released 1.3 ± 0.2 times fewer MFs than coarse SSF and achieved significantly higher overall removal efficiency (*p* < 0.05). The majority of retained MFs measured 10–50 μm in length, with effluent concentrations ranging from 1895 ± 83 MFs/L at 5 cm/h to 2993 ± 187 MFs/L at 20 cm/h.

Filtration rate also influenced turbidity and organic matter removal. Fine-sand SSF reduced turbidity by an average of 89.0% ± 3%, compared with 82.0% ± 4.0% for coarse sand, with removal efficiency declining by approximately 12.0% at 20 cm/h due to shortened water-media contact time. Effluent pH ranged between 7.4 ± 0.1 and 8.1 ± 0.1, increasing slightly with longer retention times and finer media, likely as a result of enhanced ion exchange and mineral dissolution processes. DOC concentrations decreased from 3.66 mg/L in the influent to 0.49 mg/L in the fine-sand effluent, corresponding to average removals of 35.0% ± 2 for coarse SSF and 33.0% ± 2 for fine SSF.

MFs predominantly accumulated within the upper 0–10 cm of the sand bed, highlighting the critical role of this surface layer in maintaining removal efficiency and limiting operational downtime. The observed mobility of fibres ≤ 20 μm in length, together with the measured pH range (7.0–8.5.0.5), underscores the combined influence of fibre size, media characteristics, and water chemistry on MF transport and retention behaviour.

While this study primarily focused on the physical and physico-chemical mechanisms governing MF removal, the biological component of SSF, particularly biofilm development and microbial interactions, was not explicitly investigated. Future research should address these biological processes, which are expected to further influence fibre capture, retention stability, and potential biodegradation within the filter bed.

Additional work should also evaluate SSF performance under full-scale wastewater treatment plant conditions, investigate layered or hybrid filtration configurations to balance removal efficiency with hydraulic performance, and develop monitoring and predictive tools to detect potential MF breakthrough. Finally, effective management and treatment of MF-laden wash water will also be essential to achieving fully sustainable and circular strategies for microplastic control.

## Supplementary Information

Below is the link to the electronic supplementary material.

Supplementary Material 1

## Data Availability

The authors confirm that the data supporting the findings of this study are available upon reasonable request.
