# Supplementary material for: Transport and retention of laundry microplastic fibres in slow sand filtration systems
Source: Sci Rep. 2026 Mar 6;16:14445. doi: 10.1038/s41598-026-41438-x (PMC13149595; doi:10.1038/s41598-026-41438-x)
Supplement: Supplementary file 1 — Supplementary Material 1 [file 41598_2026_41438_MOESM1_ESM.docx]

**Supplementary Information**

**Transport and retention of laundry microplastic fibres in slow sand filtration systems**

Fan Gao^1***^, Rosa Busquets^2,1**^, Luiza C. Campos^1,*^

^1^ Centre for Urban Sustainability and Resilience, Department of Civil, Environmental and Geomatic Engineering, University College London, Gower St, Bloomsbury, London, WC1E 6BT, United Kingdom

^2^ Faculty of Health, Science, Social Care and Education, School of Pharmacy and Chemistry, Kingston University, Penrhyn Road, Kingston Upon Thames, KT1 2EE, United Kingdom

* Luiza C. Campos ([l.campos@ucl.ac.uk](mailto:l.campos@ucl.ac.uk))

** Rosa Busquets ([r.busquets@kingston.ac.uk](mailto:r.busquets@kingston.ac.uk))

*** Fan Gao ([fan.gao.17@ucl.ac.uk](mailto:fan.gao.17@ucl.ac.uk))


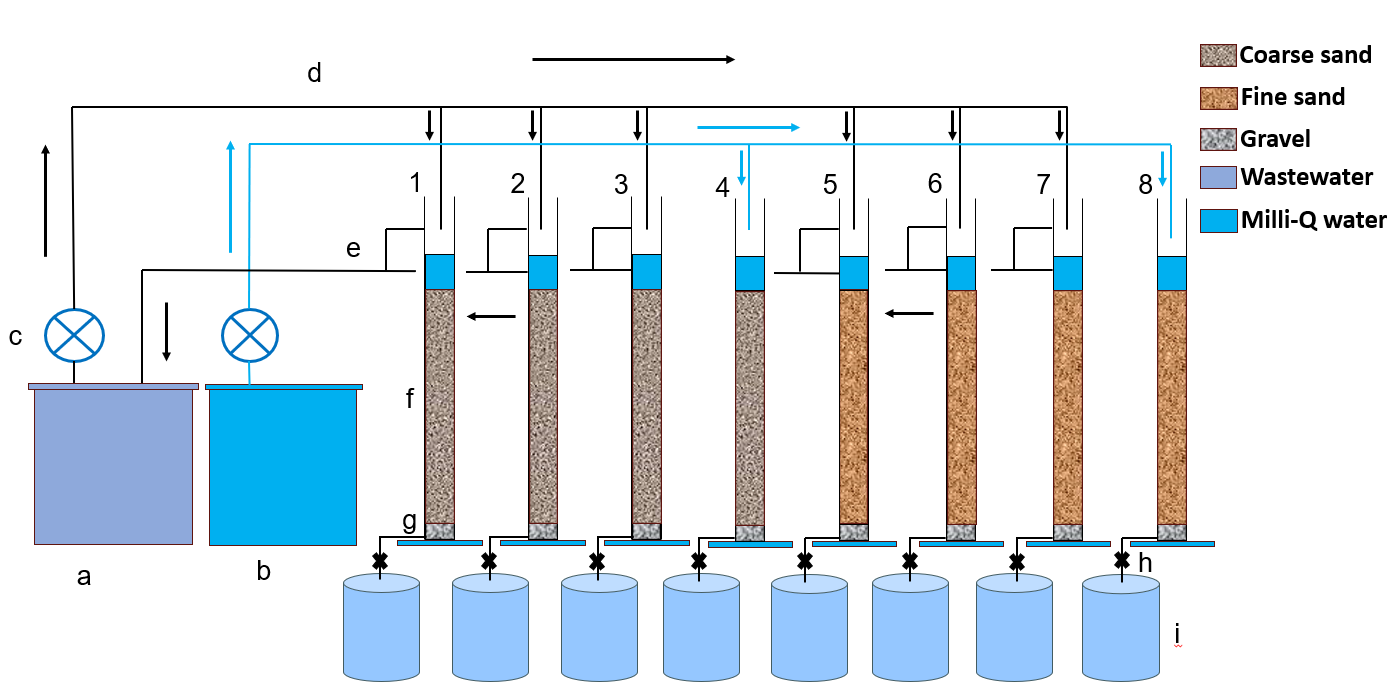


**Fig. S1** Schematic representation of the slow sand filtration experiment for the removal of microplastic fibres. The components are as follows: (a) influent tank containing laundry wastewater; (b) influent tank with ultrapure water for control; (c) peristaltic pump; (d) influent pipe; (e) overflow pipe; (f) sand filter column; (g) filter effluent pipe; (h) filter effluent valve; and (i) filter effluent collection bottle.


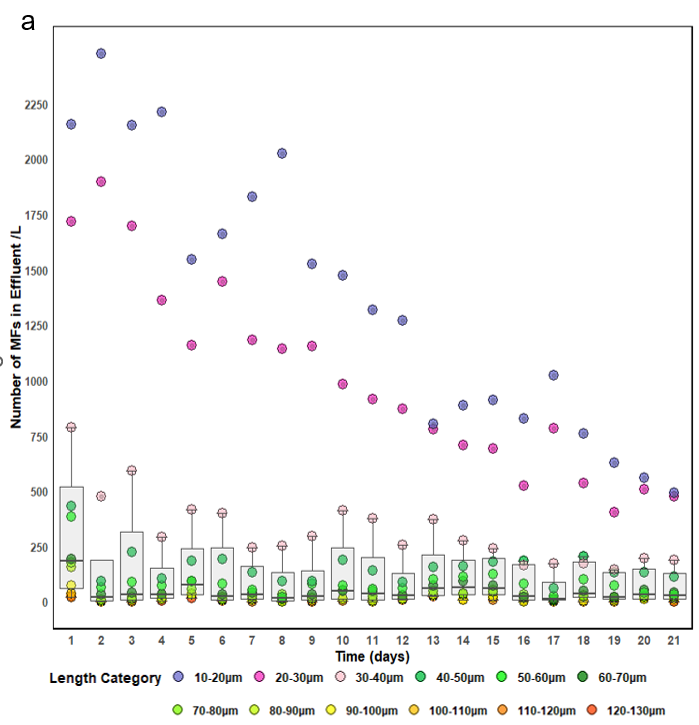


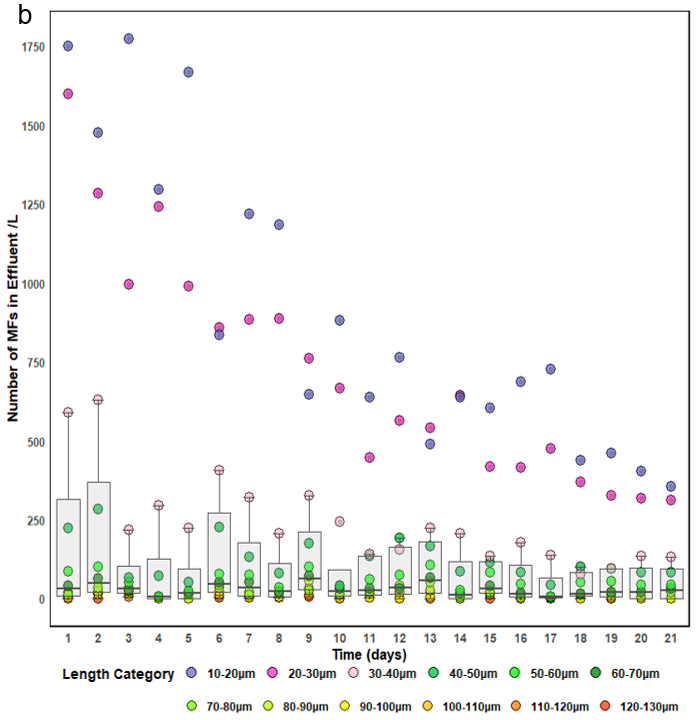


**Fig. S2** Average concentration of microplastic fibres (MFs) in the effluent of (a) coarse and (b) fine sand filters at a filtration rate of 10 cm/h during continuous operation over 21 days. The error bars represent the standard deviation from three independent experiments involving separate column packing and filtration runs. The length categories indicate the size ranges of fibres detected in the effluent.


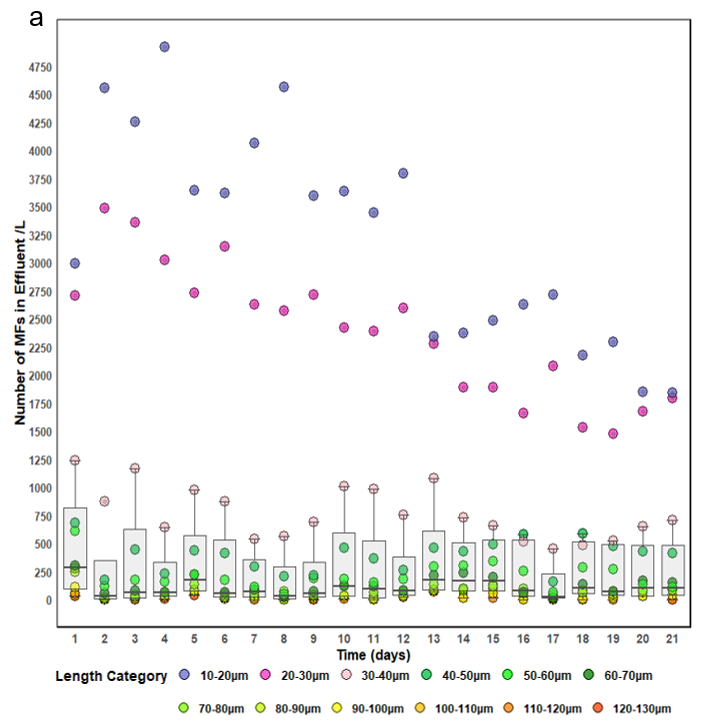


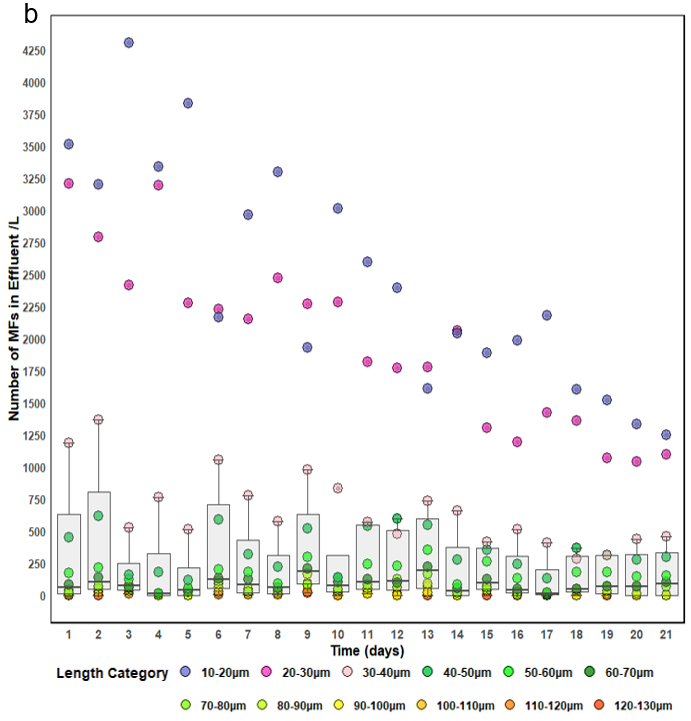


**Fig. S3** Average concentration of microplastic fibres (MFs) in the effluent of the (a) coarse and (b) fine sand filters at a filtration rate of 20 cm/h during continuous operation over 21 days. The error bars represent the standard deviation from three independent experiments involving separate column packing and filtration runs. The length categories indicate the size ranges of fibres detected in the effluent water.

a(i) a(ii) b(i)


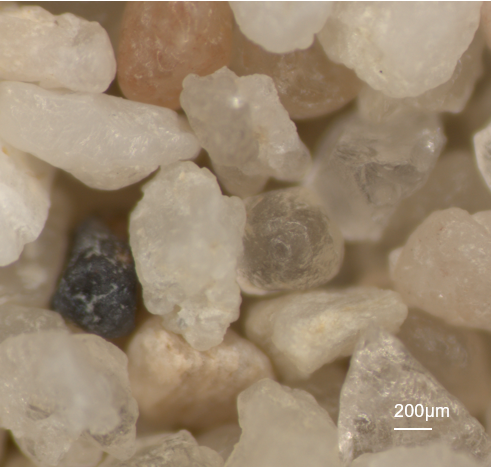

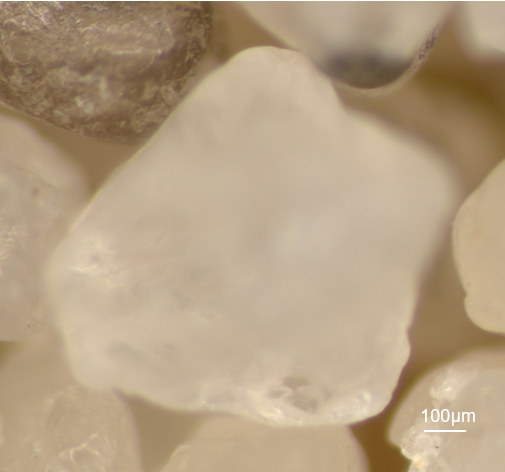

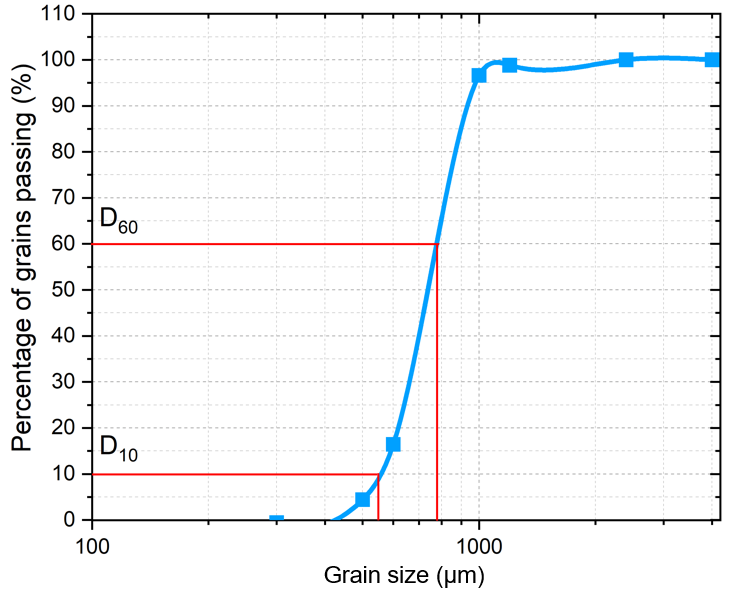


a(iii) a(iv) b(ii)


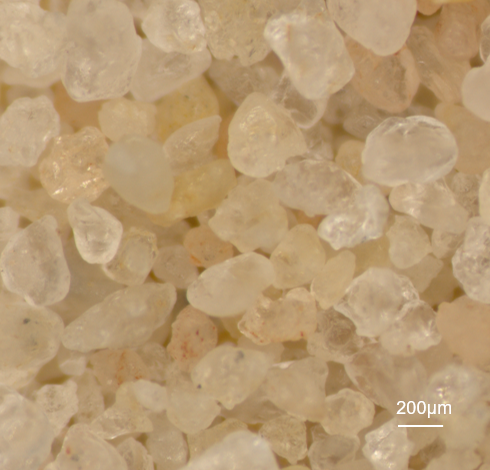

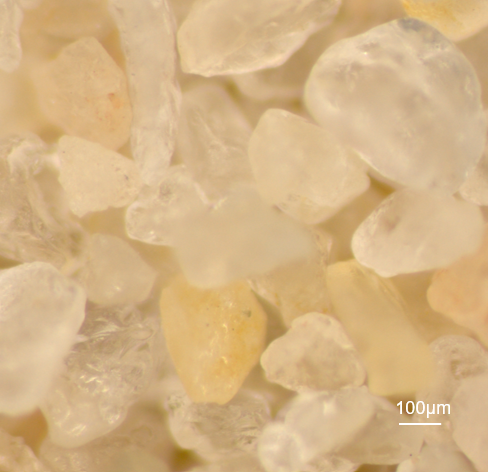

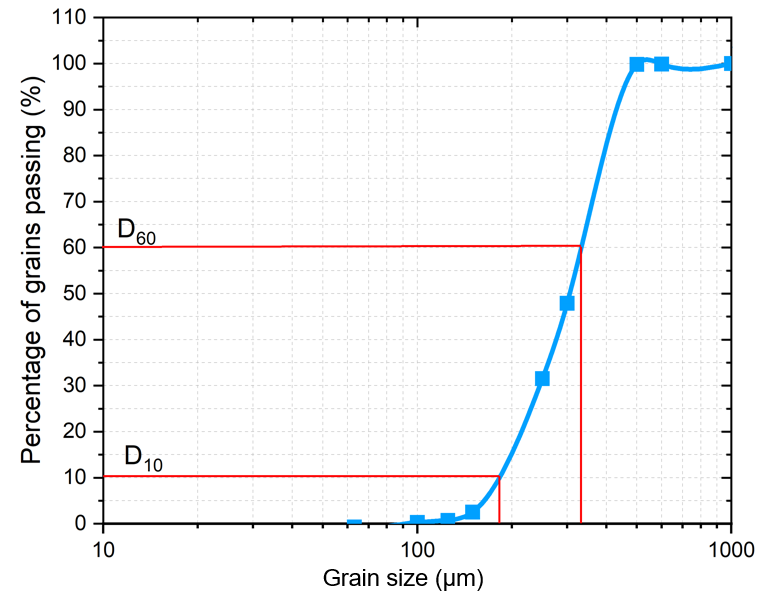


| Media characterisation | Effective size (d_10_, mm)* | d_60_, mm* | Uniformity coefficient** | Size range (mm) | Density (kg/m^3^) |
| --- | --- | --- | --- | --- | --- |
| Coarse sand | 0.60 | 0.78 | 1.4 | 0.50-1.00 | 2634 |
| Fine sand | 0.20 | 0.35 | 1.6 | 0.15-0.60 | 1538 |

**Fig. S4** Characterisation of the coarse and fine sands used in the filtration units: (a) morphology of coarse sand ((a(i)) and (a(ii))) and fine sand ((a(iii)) and (a(iv))) particles under various magnifications; (b) grain size distribution of (b(i)) coarse sand and (b(ii)) fine sand selected for the SSF system; (c) summary of characterisation information. * Effective size (d₁₀) is defined as the grain size determined from the distribution analysis at which 10% of the media (by weight) passes through; d₆₀ is the grain size at which 60% of the media (by weight) passes through. ** The uniformity coefficient is defined as the ratio of d₆₀ to d₁₀.

**Table S1** Concentration and length of microfibres in raw washing machine wastewater and in the supernatant water of the filter during the tests

|  | Raw washing machine wastewater | | | Supernatant water of filter | | | Raw washing machine wastewater | | | Supernatant water of filter | | |
| --- | --- | --- | --- | --- | --- | --- | --- | --- | --- | --- | --- | --- |
| Day | Con  (MFs/L) | SD | RSD | Con  (MFs/L) | SD | RSD | Average length (μm) | SD | RSD | Average length (μm) | SD | RSD |
| 1 | 10360 | 40 | 0.4% | 8730 | 38 | 0.3% | 35 | 3.60 | 0.1 | 34 | 2.64 | 0.1 |
| 7 | 10410 | 26 | 0.3% | 8650 | 26 | 0.3% | 34 | 2.51 | 0.1 | 33 | 4.35 | 0.1 |
| 14 | 10320 | 33 | 0.3% | 8610 | 48 | 0.5% | 36 | 2.08 | 0.1 | 34 | 2.64 | 0.1 |
| 21 | 10270 | 46 | 0.4% | 8660 | 40 | 0.4% | 35 | 3.05 | 0.1 | 35 | 1.53 | 0.1 |

Con. = concentration, SD = standard deviation, RSD = relative standard deviation, SSF = slow sand filter

**Table S2.** Average concentration of microfibres released from the top 0-10 cm of the coarse and fine sand filter beds after five rinses with 2.5 L of Milli-Q water each time.

| Concentration of MFs | Flowrate of 5 cm/h | | | Flowrate of 10 cm/h | | | Flowrate of 20 cm/h | | |
| --- | --- | --- | --- | --- | --- | --- | --- | --- | --- |
|  | Con (MFs/L) | SD | RSD | Con (MFs/L) | SD | RSD | Con (MFs/L) | SD | RSD |
| Coarse sand filter | 9463 | 18 | 0.2 | 9023 | 21 | 0.3 | 7452 | 10 | 0.1 |
| Fine sand filter | 9878 | 10 | 0.1 | 9709 | 27 | 0.4 | 8633 | 23 | 0.3 |

Con = concentration, SD = standard deviation, RSD = relative standard deviation, MFs = microfibres

**Table S3** Nitrite, nitrate, phosphate, sulphate, and ammonium concentrations in the raw washing machine wastewater

|  | Nitrite | | Nitrate | | Phosphate | | Sulphate | | Ammonium | |
| --- | --- | --- | --- | --- | --- | --- | --- | --- | --- | --- |
| Day | Con (mg/L) | RSD | Con (mg/L) | RSD | Con (mg/L) | RSD | Con (mg/L) | RSD | Con (mg/L) | RSD |
| 1 | n.d. | n.a. | 2.1 | 0.2 | 43.0 | 0.2 | 48.4 | 0.8 | n.d. | n.a. |
| 7 | n.d. | n.a. | 2.0 | 1.4 | 41.0 | 0.3 | 46.9 | 0.1 | n.d. | n.a. |
| 14 | n.d. | n.a. | 2.1 | 1.9 | 38.3 | 0.2 | 48.4 | 1.6 | n.d. | n.a. |
| 21 | n.d. | n.a. | 1.9 | 0.6 | 41.5 | 0.1 | 47.8 | 0.4 | n.d. | n.a. |

Con = concentration, RSD = relative standard deviation, n.d. = not detected, n.a. = not available

**Table S4** Nitrite, nitrate, phosphate, sulphate, and ammonium concentrations in the filter effluent during the tests

| Nitrite | Coarse SSF  Flowrate of 5 cm/h | | Coarse SSF  Flowrate of 10 cm/h | | Coarse SSF  Flowrate of 20 cm/h | | Fine SSF  Flowrate of 5 cm/h | | Fine SSF  Flowrate of 10 cm/h | | Fine SSF  Flowrate of 20 cm/h | |
| --- | --- | --- | --- | --- | --- | --- | --- | --- | --- | --- | --- | --- |
| Day | Con (mg/L) | RSD (%) | Con (mg/L) | RSD (%) | Con (mg/L) | RSD (%) | Con (mg/L) | RSD (%) | Con (mg/L) | RSD (%) | Con (mg/L) | RSD (%) |
| 1 | n.d. | n.a. | n.d. | n.a | n.d. | n.a | n.d. | n.a | n.d. | n.a. | n.d. | n.a |
| 7 | 0.02 | 1.0 | 0.01 | 1.4 | n.d. | n.a | 0.13 | 1.0 | 0.03 | 11.0 | 0.01 | 3.0 |
| 14 | 0.02 | 15.0 | 0.08 | 1.9 | 0.02 | 15.0 | 0.03 | 16.0 | 0.05 | 5.1 | 0.02 | 20.0 |
| 21 | 0.03 | 1.0 | n.d. | n.a. | 0.03 | 10.0 | 0.05 | 4.0 | n.d. | n.a. | 0.01 | 13.0 |

| Nitrate | Coarse SSF  Flowrate of 5 cm/h | | Coarse SSF  Flowrate of 10 cm/h | | Coarse SSF  Flowrate of 20 cm/h | | Fine SSF  Flowrate of 5 cm/h | | Fine SSF  Flowrate of 10 cm/h | | Fine SSF  Flowrate of 20 cm/h | |
| --- | --- | --- | --- | --- | --- | --- | --- | --- | --- | --- | --- | --- |
| Day | Con (mg/L) | RSD (%) | Con (mg/L) | RSD (%) | Con (mg/L) | RSD (%) | Con (mg/L) | RSD (%) | Con (mg/L) | RSD (%) | Con (mg/L) | RSD (%) |
| 1 | 2.1 | 20.0 | 2.1 | 20.0 | 2.1 | 20.0 | 2.1 | 20.0 | 2.1 | 20.0 | 2.1 | 20.0 |
| 7 | 3.1 | 18 | 3.0 | 17.2 | 2.8 | 17.2 | 2.8 | 10.3 | 1.7 | 23.1 | 2.6 | 24.3 |
| 14 | 5.7 | 5.9 | 5.9 | 12.4 | 4.1 | 2.4 | 3.6 | 6.8 | 1.6 | 6.5 | 2.5 | 9.9 |
| 21 | 8.2 | 5.5 | 7.4 | 7.5 | 5.8 | 6.1 | 2.6 | 5.1 | 1.1 | 7.8 | 2.7 | 11.2 |

| Phosphate | Coarse SSF  Flowrate of 5 cm/h | | Coarse SSF  Flowrate of 10 cm/h | | Coarse SSF  Flowrate of 20 cm/h | | Fine SSF  Flowrate of 5 cm/h | | Fine SSF  Flowrate of 10 cm/h | | Fine SSF  Flowrate of 20 cm/h | |
| --- | --- | --- | --- | --- | --- | --- | --- | --- | --- | --- | --- | --- |
| Day | Con (mg/L) | RSD (%) | Con (mg/L) | RSD (%) | Con (mg/L) | RSD (%) | Con (mg/L) | RSD (%) | Con (mg/L) | RSD (%) | Con (mg/L) | RSD (%) |
| 1 | 43.0 | 20.0 | 43.0 | 20.0 | 43.0 | 20.0 | 43.0 | 20.0 | 43.0 | 20.0 | 43.0 | 20.0 |
| 7 | 22.2 | 18.3 | 19.3 | 10.2 | 22.4 | 10.2 | 2.0 | 13.6 | 4.6 | 5.3 | 6.8 | 15.9 |
| 14 | 3.1 | 16.6 | 6.6 | 18.4 | 7.5 | 18.3 | 1.9 | 10.0 | 3.9 | 11.9 | 5.2 | 13.6 |
| 21 | 2.5 | 5.9 | 5.9 | 1.7 | 6.9 | 6.6 | 0.8 | 5.1 | 2.2 | 7.5 | 3.7 | 3.6 |

| Sulphate | Coarse SSF  Flowrate of 5 cm/h | | Coarse SSF  Flowrate of 10 cm/h | | Coarse SSF  Flowrate of 20 cm/h | | Fine SSF  Flowrate of 5 cm/h | | Fine SSF  Flowrate of 10 cm/h | | Fine SSF  Flowrate of 20 cm/h | |
| --- | --- | --- | --- | --- | --- | --- | --- | --- | --- | --- | --- | --- |
| Day | Con (mg/L) | RSD (%) | Con (mg/L) | RSD (%) | Con (mg/L) | RSD (%) | Con (mg/L) | RSD (%) | Con (mg/L) | RSD (%) | Con (mg/L) | RSD (%) |
| 1 | 48.4 | 80.0 | 48.4 | 80 | 48.4 | 80.0 | 48.4 | 80 | 48.4 | 80.0 | 48.4 | 80.0 |
| 7 | 43.9 | 27.3 | 47.5 | 11.9 | 45.8 | 80.3 | 40.9 | 20.7 | 41.6 | 27.0 | 44.0 | 12.1 |
| 14 | 44.6 | 19.6 | 45.6 | 21.0 | 44.1 | 11.9 | 41.8 | 79.3 | 46.2 | 8.5 | 44.7 | 16.8 |
| 21 | 39.5 | 8.5 | 41.7 | 43.5 | 43.6 | 21.1 | 31.7 | 16.8 | 38.8 | 16.2 | 41.3 | 11.5 |

| Ammonium | Coarse SSF  Flowrate of 5 cm/h | | Coarse SSF  Flowrate of 10 cm/h | | Coarse SSF  Flowrate of 20 cm/h | | Fine SSF  Flowrate of 5 cm/h | | Fine SSF  Flowrate of 10 cm/h | | Fine SSF  Flowrate of 20 cm/h | |
| --- | --- | --- | --- | --- | --- | --- | --- | --- | --- | --- | --- | --- |
| Day | Con (mg/L) | RSD | Con (mg/L) | RSD | Con (mg/L) | RSD | Con (mg/L) | RSD | Con (mg/L) | RSD | Con (mg/L) | RSD |
| 1 | n.d. | n.a. | n.d. | n.a. | n.d. | n.a. | n.d. | n.a. | n.d. | n.a. | n.d. | n.a. |
| 7 | n.d. | n.a. | n.d. | n.a. | n.d. | n.a. | n.d. | n.a. | n.d. | n.a. | n.d. | n.a. |
| 14 | n.d. | n.a. | n.d. | n.a. | n.d. | n.a. | n.d. | n.a. | n.d. | n.a. | n.d. | n.a. |
| 21 | n.d. | n.a. | n.d. | n.a. | n.d. | n.a. | n.d. | n.a. | n.d. | n.a. | n.d. | n.a. |

Con = concentration, SD = standard deviation, RSD = relative standard deviation, SSF = slow sand filter, n.d. = not detected, n.a. = not available
